# The Effects of Fire Severity on Macroinvertebrate Detritivores and Leaf Litter Decomposition

**DOI:** 10.1371/journal.pone.0124556

**Published:** 2015-04-16

**Authors:** Sebastian Buckingham, Nick Murphy, Heloise Gibb

**Affiliations:** 1 Department of Zoology, La Trobe University, Melbourne, VIC, 3086, Australia; 2 Department of Genetics, La Trobe University, Melbourne, VIC, 3086, Australia; Roehampton university, UNITED KINGDOM

## Abstract

High severity wildfire events are a feature of forests globally and are likely to be more prevalent with climate change. As a disturbance process, fire has the potential to change important ecological functions, such as decomposition, through its impact on biodiversity. Despite the recognised importance of decomposition in terms of fuel loads and energy flow, little is known about the post-fire effects of fire severity on decomposition by litter-dwelling macroinvertebrate detritivores. We tested the hypotheses that: 1) increasing fire severity is associated with decreased rates of leaf litter decomposition by macroinvertebrate detritivores; and 2) the abundance and biomass of macroinvertebrate detritivores decreases with increasing fire severity, while body size increases. We used a litterbag experiment at long-unburnt, ground-burnt and crown-burnt sites (n = 7 for all treatments) to test the effect of fire severity on: a) macroinvertebrate-driven break-down of litter fuel loads; and b) the size and abundance of macroinvertebrate detritivores three years after fire. Microhabitat conditions differed among fire severity classes. Macroinvertebrate exclusion reduced litter decomposition by 34.7%. Macroinvertebrate detritivores were larger and less abundant following higher severity fires, possibly as a result of fire-induced changes in habitat structure. Opposing effects of fire severity on macroinvertebrate abundance and body size resulted in both similar detritivore biomass and, most interestingly, no differences in leaf litter decomposition under different fire severities. This suggests that the diversity of macroinvertebrates enhances functional resilience of litter decomposition to fire and that litter-breakdown is not inhibited within three years following a high severity fire in this forest type and where recolonisation sources are readily available. We found no support for the hypothesis that high severity fires reduce litter decomposition and therefore increase the likelihood of future fires.

## Introduction

Fire is recognised globally as an important form of disturbance due to its widespread occurrence and potential to alter the structure and function of ecosystems [1]. High severity wildfire events occur in forests exposed to persistent high pressure system events that dry fuel [2]. Wider fluctuations in weather conditions, periods of increased dryness extending fire seasons and more regular extreme hot dry winds are thought to be more likely under climate change, enhancing the likelihood of severe fires [3,4]. Although numerous studies have examined the responses of biodiversity and ecosystem function to fire frequency (e.g. [5–11], fewer studies have examined the effect of fire severity on biota [12–16].

In southern Australian forests, litter from trees is the defining factor driving fire regimes [17], and plays a large role in contributing to fire severity and rate of spread [18–20]. Due its role as fuel, the build-up of litter plays a significant role in assessing bushfire hazard and fuel reduction burns are usually targeted to reduce litter [21]. The balance between litter fall and decomposition is therefore central to the prevention of fuel build-up. Globally, decomposition can be explained in terms of three major components: climatic conditions, leaf litter quality and biota [22,23]. At local scales, where single tree species often dominate, climate and litter quality are likely to vary less, increasing the relative importance of biota in decomposition. Leaf litter decomposition occurs through chemical change caused by the action of microbes or the physical fragmentation of leaves through consumption of leaf material by macroinvertebrate detritivores, resulting in acceleration of microbial activity (Wardle 2002). Leaf fragmentation is potentially a major driver of decomposition in tropical and temperate climates where conditions such as moisture and temperature are less limiting for macroinvertebrates [24–26]. At local scales, the role of other drivers, such as climate and leaf substrate quality may be far less predictive of litter decomposition than measures of the macroinvertebrate detritivore assemblage [22,25,26]. Two recent meta-analyses showed that litter quality and climate are important drivers of decomposition [25,26]. However, these studies also emphasised that macroinvertebrate detritivores had substantial effects on decomposition and that this third factor has been neglected and warranted emphasis in future studies. This study responds to this reported oversight.

Disturbance may change the rate of decomposition by altering the characteristics of litter-feeding macroinvertebrate assemblages [22,27,28]. Previous studies suggest that macroinvertebrate diversity and function are affected by disturbance due to fire [11,29]. Fire is characterised by its frequency, extent and severity [30] and each of these elements may affect outcomes for macroinvertebrate assemblages and the functions they perform. Fire severity describes the loss of or change in organic matter aboveground and belowground following a fire [31]. High severity fires have previously been shown to have long-lasting effects on vegetation recovery [32] and to reduce the abundance and species diversity of fungi [33] and microinvertebrates [16,34], while bird assemblages respond differently to fires of differing severity [35]. Reduced litter decomposition by macroinvertebrates is associated with increased fire frequency [36]. However, the effects of fire severity on macroinvertebrate biodiversity and function are less well known [12,14,37].

High severity or crown fires present macroinvertebrates with particular challenges. Entire oxidation of organic matter of surface soil and logs occurs in high severity forest fires [19,38], resulting in the complete loss of habitat for litter-feeding macroinvertebrates, compared with more patchy low severity fires. Wikars and Schimmel (2001) [12] showed that, in severe fires, invertebrates living deeper in soil suffered reduced mortality compared with those in the vegetation and litter layers. However, no studies have examined the effects of fire severity on macroinvertebrate-driven decomposition. The loss of habitat following fire has immediate and ongoing effects for fauna populations [39]. After severe fire, fauna populations and their functional roles could be affected by local extinction and their capacity for local recruitment, loss of habitat complexity, and altered species interactions, including competition and predation [40,41]. Despite the key functional role of macroinvertebrates in litter decomposition [22], no studies have examined the effects of fire severity on macroinvertebrate-driven decomposition for leaf litter, although one study has examined the effects of fire severity on the decomposition of cellulose [42]. A closer examination of functional responses is required if we are to explain mechanisms driving change in the functional importance of macroinvertebrate detritivores [24–26].

We tested the effect of fire severity on macroinvertebrate abundance and body size and litter break-down in Eucalypt forests in south-eastern Australia that had experienced the catastrophic ‘Black Saturday’ fires in 2009. We used a manipulative experiment to test the following hypotheses: 1) increasing fire severity is associated with decreased rates of leaf litter decomposition by macroinvertebrate detritivores; 2) the abundance and biomass of macroinvertebrate detritivores decreases with increasing fire severity, while body size increases.

## Methods

### Study sites

This research was conducted at Kinglake National Park, Mount Robertson, Toolangi and Marysville State Forests with the permission of the Department of Sustainability and Environment, Victoria, Australia (Permit Number: 10005924). Field studies did not involve any threatened or endangered species.

This study was carried out in forests located within the 2009 Kilmore East-Murrindindi fire complex, in the foothills of the Great Dividing Range, Victoria, southeast Australia (37° 34'S, 145° 30'E) (S1 Fig). The climate is temperate with a mean annual rainfall of 1373 mm recorded at 595 m a.s.l. The mean daily maximum summer temperature is 23.2°C, while the mean daily minimum winter temperature is 3.8°C. Sites selected were located in damp sclerophyll forest in gullies dominated by messmate stringybark (*Eucalyptus obliqua)*, around 25 m tall with a dense midstorey of tree fern (*Dicksonia antarctica*) and hazel pomaderris (*Pomaderris aspera)*. Other tree species that occurred in the vicinity of sites but were not dominant included blue gum (*Eucalyptus globulus*), mountain grey gum (*Eucalyptus cypellocarpa*) or small-leaved peppermint (*Eucalyptus radiata*). These damp gullies are typically surrounded by dry sclerophyll forest with an open midstorey [43]. Soils were clay loam and sandy clay loam derived from alluvial and/or colluvial weathering. Aspect and slope varied among sites (S1 Table).

The fire began on February 9^th^, 2009, as two separate fires at Kilmore East and Murrindindi and burnt to the east under severe weather conditions for the first 12 hours, during which time most of the high severity crown fires had occurred (S1 Fig). The two fires later joined under lower wind speeds and humidity to cover an area of over 228,000 ha [19,44,45]. Three fire severities were selected in this study and refer to the severity of the fire that occurred in February 2009 [19,31]. Fire severities were classified as follows: 1) Crown burnt (highest severity), referring to burning of all leaves in the tree crown and midstorey, leaf litter, humus, and logs; 2) Ground burnt (lower severity), referring to the incineration or charring of the litter layer and underlying humus layer; logs are typically charred where exposed but sometimes wholly burnt and the shrub and groundlayer vegetation, but not the tree crown, is typically burnt; and 3) Unburnt, referring to sites not burnt or directly affected by fire in 2009. All sites were unburnt and unaffected by logging for at least 20 years prior to 2009.

### Litter decomposition

We commenced with two decomposition experiments in spring 2011. Sites were selected in the three severity classes with seven replicates making a total of 21 sites interspersed over a 30 by 50 km area (S1 Fig). Sites were separated by a minimum distance of 150 m and sites of the same fire severity were separated by a minimum of 1.15 km. Unburnt sites ranged 10 to 20 m from the burnt edge. For the first experiment, we placed litterbags directly on the soil and covered them with leaf litter at each site. Litter bags were 180 mm x 180 mm in dimensions and contained 10 g of dried *E*. *obliqua* leaves (collected green). Green leaves make up around 10% of total leaf fall in *E*. *obliqua* forest [46], so are a representative substrate, but differ chemically from senescent leaves [47]. Although this is a small proportion of the total litter fall, using green leaves allowed us to standardise the litter bags across all sites. Senescent leaves or 'leaves on strings' were used to verify that any artificiality in the methods used in the litter bag experiment due to leaf age did not affect the relative rates of decomposition among fire severity treatments. Litter quality and climate are important drivers of decomposition [25,26], but this study did not aim to address these issues. Litter quality was controlled for by using a controlled experiment, with litter sourced from the same locations and tree species. Climate was controlled for by using a limited geographic area for the study.

Litter bags were dried in ovens at 60°C for 3 days and weighed before and after the field experiment. Frass (excreted organic material less than 2 mm diameter) and soil debris were removed from leaves after the experiment with the aid of a 3 mm sieve. In order to determine the role of macroinvertebrate detritivores in the first experiment, we used a “control” treatment (n = 10 bags per site, unmanipulated) to measure the effects of fauna and microbes on decomposition and an “insecticide” treatment (n = 10 bags per site, macroinvertebrates excluded) to measure the effects of microbes only on decomposition rates. The litter bags had a mesh size of 8 mm to allow access by most macroinvertebrate detritivores, microarthropods, fungi and bacteria. Before placement in the field, we soaked the “insecticide” litter bags in a pyrethroid (Bifenthrin 10 mg/L) solution for the insecticide treatment and the control was placed in water only, both for 1 hour. Chemical exclusion using a pyrethroid was preferred over a physical exclusion using fine mesh because no studies using that method have reported success in excluding moth larvae (Lepidoptera) in Australia [36,48]. Pyrethroids do not affect the function of fungi and bacteria [49]. In the first experiment, litter bags were placed along a 30 m x 2 m belt transect at each site. The litter bag treatments (2 control and 2 insecticide) were located at each station along the transect at 3 m intervals (10 stations and 40 litter bags per site). Control and insecticide litter bags were 2 m apart at each station. Ten control and ten insecticide treatment litter bags were removed from each site and measured at 6 months (autumn 2012) and the remaining ten control and ten treatment litter bags were removed from each site at 12 months (spring 2012). A total of 840 litter bags were placed in the field (10 replicates x 2 treatments x 2 sampling periods x 21 sites).

We used a second method to determine levels of leaf breakdown because conditions within litter bags can potentially alter decomposition rates [50,51]. Here, we tied five senescent leaves to a piece of nylon string placed 50 cm from the control litterbag at six of the ten stations across 21 sites for a total of 186 leaf area subsamples. This experiment presented more realistic conditions for decomposition: senescent leaves scattered amongst the leaf litter. However, unlike the litter bag experiment, it did not allow us to sample the decomposer assemblage. We measured leaf area before placement in the field in spring 2011 and after 12 months using an *Area Meter AM 300* (Bioscientific, Australia), then calculated percentage area loss. Leaves were placed in contact with the soil. As well as providing a more ‘natural’ microclimate, leaves tied to strings may provide better access to larger fauna. However, they could also allow larger fragments to break off and become lost. The use of both litter bag and ‘leaves on string’ methods should provide estimates that bound true values of leaf breakdown by macroinvertebrate detritivores [52]. If the area loss from the ‘leaves on string’ experiment and mass loss from the litter bag experiment are related, this should indicate that litter bag experiment provided a suitable model for responses to senescent leaves.

### Macroinvertebrate detritivores

To determine the abundance, size and biomass of macroinvertebrate detritivores, litter bags (control and insecticide) were placed in Tullgren funnels with fluorescent 30 watt light bulbs within 24 hours of collection and were removed from funnels after 48 hours. Earlier tests using 10 grams of wet leaf litter collected from the field confirmed that extraction of all living animals was achieved over a 2 day period. Mesh size of 10 mm permitted movement of macroinvertebrates into funnels and vials filled with 70 percent ethanol.

We considered only macroinvertebrate detritivores 2 mm or greater in length for this analysis because they included all the functionally important fauna (principally larvae) that ingest leaf litter [52]. Macroinvertebrate detritivores were identified to family and morphospecies. Morphospecies were assigned to the following functional groups: detritivores, fungivores and predators using references [53–55] and a dissection microscope. Abundance and body length were recorded for all individuals. Biomass was calculated from body length-mass relationships Saint-Germain (2007) [56] using algorithms from Hodar (1996) [57] for all taxa with the exception of amphipods for which we used Gruner (2203) [58]. A subsample of macroinvertebrate detritivores were measured for length, then dried and weighed to confirm that algorithms accurately estimated their biomass. We used the mean value of abundance, size and biomass at each site to test changes in the assemblage.

### Microhabitat

Nine microhabitat measurements were made for each of the control and insecticide litterbags making a total of 20 measurements per site during the autumn 2012 leaf litterbag collection. Percentage cover of foliage was the total of ground, midstorey and canopy cover. Ground cover was estimated visually within a 30 cm x 30 cm quadrat. Midstorey and canopy cover were estimated within a 2 m x 2 m quadrat. Percentage cover of leaf litter, bare ground and moss was estimated visually within a 30 cm x 30 cm quadrat. Soil disturbance was the visual estimation of the percentage of soil covering litterbags from foraging vertebrates e.g. superb lyrebirds (*Menura novaehollandiae*) and long-nosed bandicoots (*Perameles nasuta*). Soil included organic and mineral components. Litter depth was measured at three points within each quadrat. The distance to the nearest log of diameter greater than 10 cm was measured. Disturbance was recorded as the percentage cover of soil covering each litterbag principally caused by digging of ground foraging vertebrate species. Moisture was measured with a meter *CT-250 Cool Tech* (Testequipment, Australia) inserted within the litterbag and relative humidity was recorded by placing *Kestrel 4500 Pocket Weather Tracker* (Nielsen-Kellerman, U.S.A.) on the soil beneath each litter bag immediately after collection.

### Statistical analysis

A principal component analysis on correlations was carried out on the nine microhabitat predictor variables, and a biplot produced using packages MASS and Calibrate in R (R Development Core Team 2012 [59,60]). The purpose of this analysis was to create a multidimensional presentation of the variability of microhabitat conditions in relation to sites, and to form the basis of a principal component regression (PCR) for leaf litter decomposition and macroinvertebrate measures (abundance, size and biomass). Axes that best described the data set were originally to be selected for the PCR because microhabitat variables were likely to covary [61]. However, after examining the eigen values against the broken stick model (i.e. components that explain the vast proportion of the variability), the number of principal components equalled the majority of the original microhabitat variables, so PCR was not used.

We used ordinary least squares (OLS) regression to test the effect of fire severity, the experimental treatment and their interaction, and nine microhabitat variables on the mean and variance per site of mass loss from the litter bag experiment and the leaf area lost per site from the leaves on string experiment after 12 months. Variance of mass lost is indicative of heterogeneity of decomposition within sites. OLS regression was used to test the effect of fire severity, the experimental treatment and their interaction on the mean mass at six months to determine if there were differences in decomposition halfway through the experiment. Results were similar at 6 and 12 months with 1 year showing the full effects of the experiment therefore we used 12 month data to test against predictor variables. OLS regression was used to test the effect of fire severity and nine microhabitat variables on the abundance, size and biomass of macroinvertebrate detritivores per site at six months (the midpoint in the experiment). OLS is used to minimise the distances in the observed responses with those predicted in the linear approximation. An AIC model-stepwise regression was used to determine the best predictor variables automatically in all six tests. To confirm whether the control for mass loss was measuring an approximation of the real function of macroinvertebrate detritivores; we used an OLS regression to test for a positive relationship between mass loss (control) and leaf area loss at 12 months. To compare microhabitat variables between three fire severities, we used ANOVA (Analysis of Variance) tests. We used a generalised linear model (GLM) with a negative binomial function (using package MASS in R) to test the effects of fire severity on moss cover because this best fitted the response distribution i.e. the high proportion of zero values did not fit the assumptions of ANOVA in this case. Post-hoc Tukey's tests were conducted when the effect of fire severity was significant.

## Results

### Microhabitat

**Fig 1:**
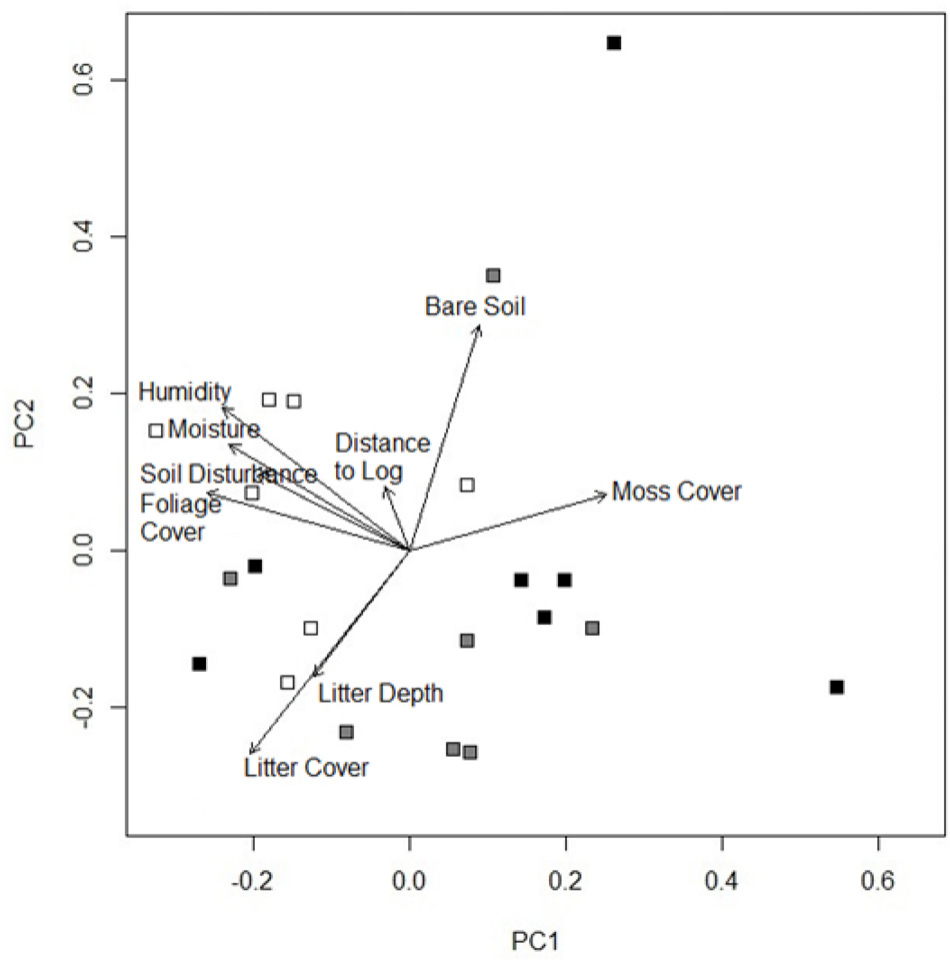
Principal components analysis of microhabitat variables at 21 sites from three different fire regimes (Unburnt, open; Ground Burnt, grey; Crown Burnt, black). Vectors show the strength and direction of the relationship between the microhabitat variables and axes. PC1 and PC2 explained 30 and 22% of the variance in microhabitat characteristics. The values for the microhabitat variables in relation to the two axes, principal component one (PC1) and principal component two (PC2), are listed in Table 1.

**Fig 2:**
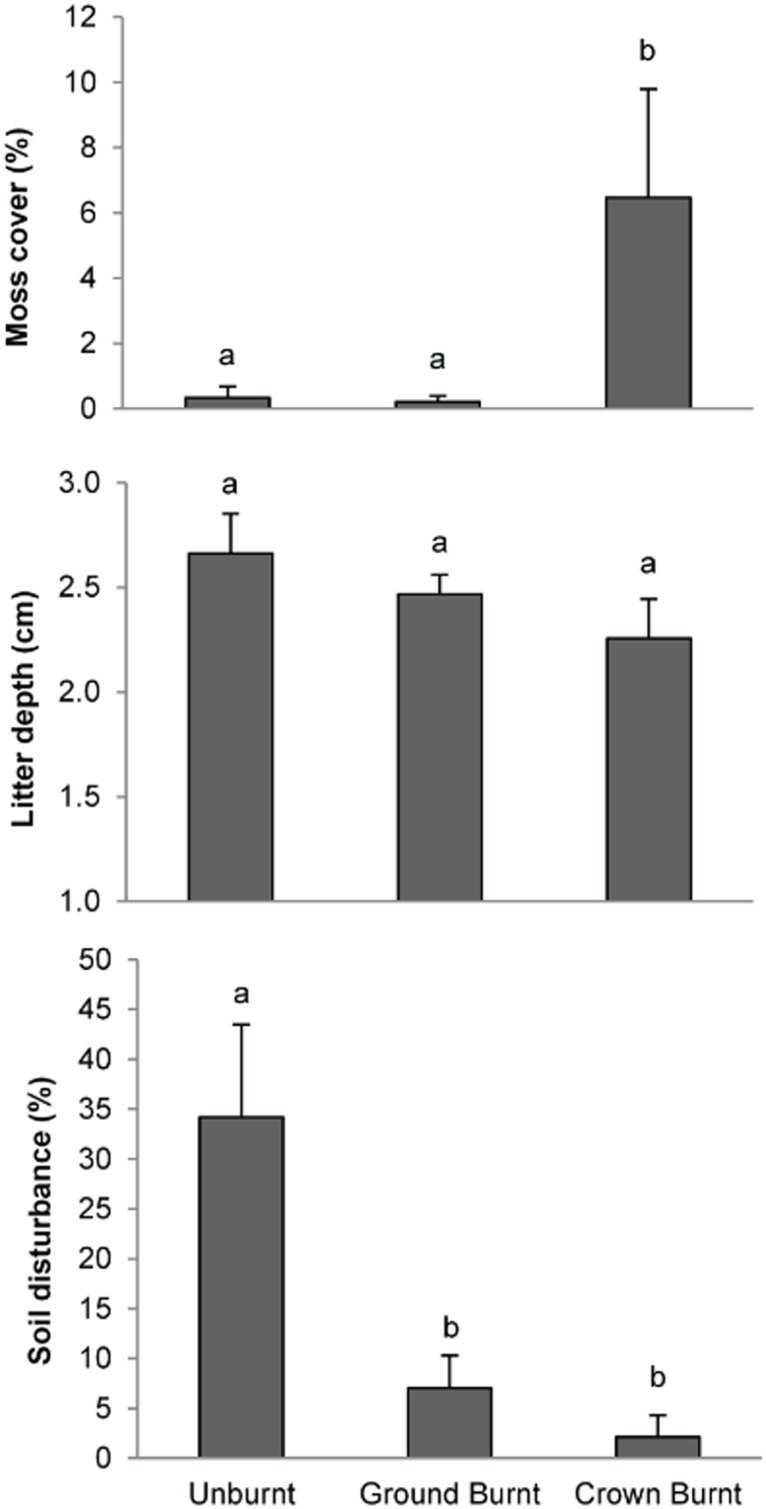
Mean ± SE of: a) moss cover; b) litter depth; and c) soil disturbance percentage for three fire severities. Different letters indicate values were significantly different in post-hoc tests.

Principal components analysis of microhabitat revealed distinct patterns with respect to microhabitat variables and fire severity even three years after fire (Fig 1). Principal component one (PC1) and principal component two (PC2) represent 30 and 22% of microhabitat variability. PC1 was positively correlated with moss cover and negatively correlated with foliage cover, moisture, relative humidity, litter cover and soil disturbance (Fig 1; Table 1). Crown burnt sites were high on PC1, while unburnt sites were low on this axis. PC2 was positively correlated with bare soil and moisture and negatively correlated with litter cover and litter depth. There was no clear trend for sites along the PC2 axis. Moss cover was greatest in crown burnt sites (F_(2,18)_ = 7.96, P<0.001), while soil disturbance was greatest in unburnt sites (F_(2,18)_ = 8.83, P<0.01). Litter depth, an important measure of fuel availability, did not respond to fire severity (F_(2,18)_ = 1.56, P = 0.24), (Fig 2).

**Table 1:** Principal component analysis (PCA) of microhabitat variables.

Microhabitat Variable	PC1	PC2
Foliage Cover	**0.44**	0.15
Moss Cover	**0.43**	0.14
Moisture	**0.41**	**0.36**
Relative Humidity	**0.39**	0.27
Litter Cover	**0.35**	**0.51**
Soil Disturbance	**0.33**	0.20
Litter Depth	0.21	**0.32**
Bare Soil	0.15	**0.57**
Log Distance	0.05	0.16

Eigenvalues were 2.7 and 2.0 for PC1 and PC2 respectively, explaining 30 and 22% of the variance in microhabitat characteristics, respectively. Variables with values greater than 0.3 are considered to contribute strongly to the axes and are represented in bold.

### Litter decomposition

**Fig 3:**
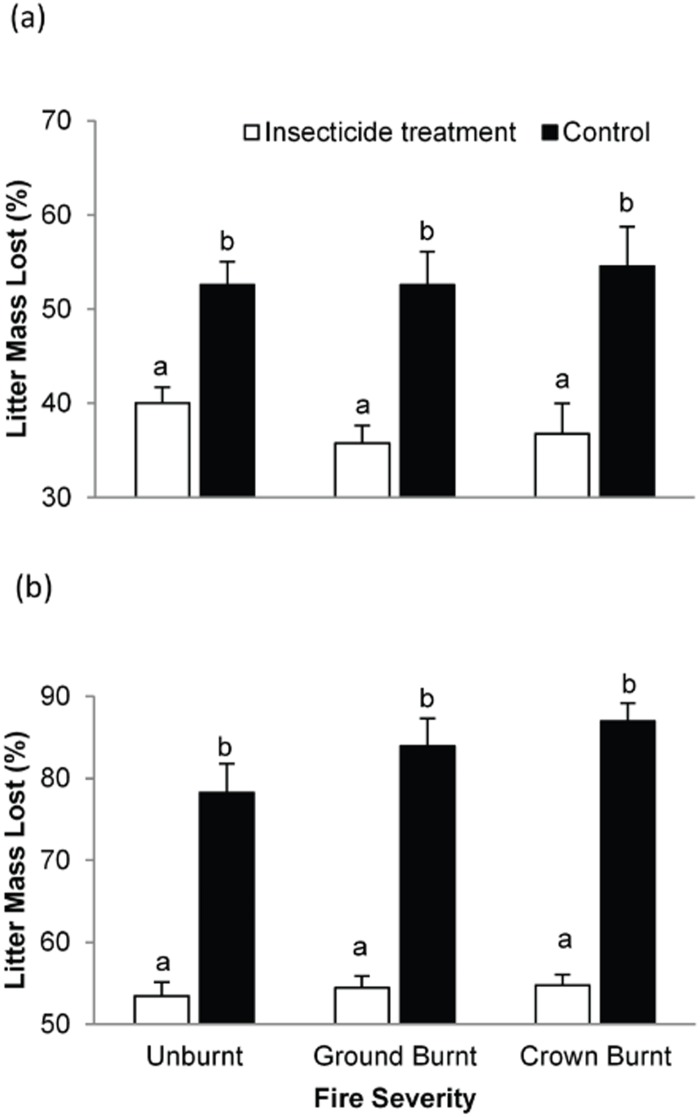
Mean ± SE of leaf litter mass lost in insecticide treatment and control litter bags under different fire severities after: a) six; and b) twelve months. Different letters indicate values were significantly different in post-hoc tests.

The regression testing the relationship between mean leaf area loss of senescent leaves and mass loss from green leaf litterbags was significant, (R^2^ = 0.18, F_(1,19)_ = 5.36, P<0.032), confirming that these measures showed similar response patterns. For the litter bag experiment, mass loss was greater for the control than for the insecticide treatment, in agreement with predictions. Litterbags treated with insecticide lost 29.5% and 34.7% less mass than control litterbags at 6 months (autumn) and 12 months (spring) respectively, (Fig 3). Macroinvertebrate detritivore presence was the single most important determinant of mass lost and was highly significant (Table 2). We expected that high severity fires would inhibit the functional role of macroinvertebrate detritivore in leaf litter loss. However, the effect of fire severity on mean mass loss (control) was not significant; instead, there was a positive trend with fire severity for leaf mass loss (control) in both whole and best fit models (Table 2, Fig 3b). The whole and best fit models testing the effect of fire severity and microhabitat on mean leaf area loss were not significant (Table 2). There was no significant interaction between insecticide treatment and fire severity, suggesting that macroinvertebrates were responsible for a similar proportion of decomposition, independent of fire severity (Table 2). Moisture and relative humidity (a covariate measure) were the only predictors with strong and consistent positive effect on mean decomposition rates when macroinvertebrates were present, and only moisture had a significant positive effect. Mass loss variance (i.e., heterogeneity in decomposition within sites) did not respond significantly to fire severity (Table 2), but was significantly affected by litter depth in the whole model and litter depth and soil disturbance in the best model.

**Table 2:** OLS regression testing on the effect of fire severity, insecticide treatment and nine microhabitat variables on the mean and variance of leaf mass lost (litter bag experiment) and the mean of leaf area lost (leaves on string experiment) at 12 months.

	Leaf mass loss (mean)				Leaf area loss (mean)				Leaf mass loss (variance)			
	Whole model d.f. = 11,27		Best fit model d.f. = 5,36		Whole model d.f. = 11,9		Best fit model d.f. = 7,13		Whole model d.f. = 14,27		Best fit model d.f. = 9,32	
	F-value	P	F-value	P	F-value	P	F-value	P	F-value	P	F-value	P
**Fire Severity**	2.45	(+)	2.53	(+)	1.71	(+)	2.14	(+)	0.03	(+)	0.03	(+)
**Treatment**	236.42	(-)***	243.02	(-)***					0.43	(-)	0.45	
**Foliage Cover**	0.53	(-)	0.55	(-)	4.73	(-)	5.95	(-)*	0.59	(+)	0.63	(+)
**Bare Soil**	0.12	(-)	0.12	(-)	4.00	(-)	4.98	(-)*	0.09	(-)		
**Moss Cover**	1.51	(-)			4.59	(-)	5.77	(-)*	0.07	(-)		
**Litter Cover**	0.31	(-)			0.33	(-)			1.31	(-)	0.36	(-)
**Litter Depth**	0.61	(-)	0.39	(-)	0.87	(-)			7.13	(+)*	6.40	(+)*
**Soil disturbance**	0.10	(-)			1.43	(-)			3.61	(+)*	4.42	(+)*
**Moisture**	7.67	(+)*	7.89	(+)**	6.03	(+)*			0.87	(-)		
**Relative Humidity**	0.96	(+)			7.70	(+)*	19.16	(+)***	0.19	(+)		
**Distance to Log**	1.38	(+)			8.57	(+)*	10.48	(+)**	0.62	(-)		
**Fire Severity:Treatment**	0.28	(+)	1.38	(+)					0.23	(+)	0.24	(+)

For the leaves on string experiment, the whole model test of leaf area loss also showed a significant positive response to moisture and relative humidity and distance to log. The best fit model also indicated significant negative responses for foliage cover, bare soil and moss cover (Table 2).

### Macroinvertebrate detritivores

**Fig 4:**
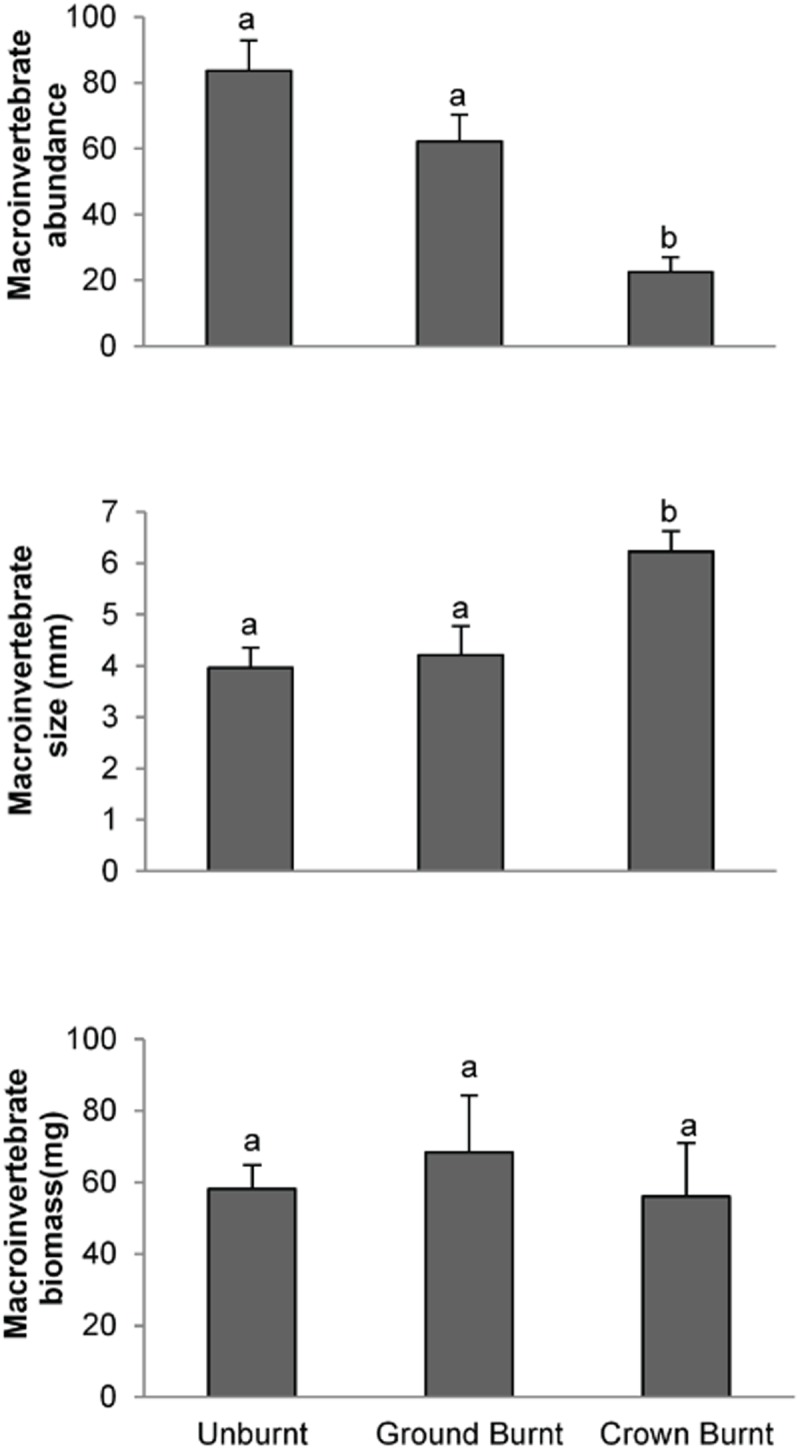
Mean ± SE of macroinvertebrate detritivore: a) abundance; b) size and c) biomass under different fire severities. Different letters indicate values were significantly different in post-hoc tests.

Macroinvertebrate detritivore size and abundance differed, depending on fire severity. The abundance of macroinvertebrate detritivores differed between fire severity classes, with fewer collected from crown burnt sites (Table 3, Fig 4). In contrast, macroinvertebrate detritivore size (measured as site means of individuals) was greatest in crown burnt sites, however, interestingly macroinvertebrate detritivore biomass did not differ significantly between fire severities.

**Table 3:** OLS regression testing the effect fire severity and nine microhabitat variables on three macroinvertebrate detritivore traits (abundance, size and biomass) from the litter bag experiment.

	Abundance				Size				Biomass			
	Whole model d.f. = 11,9		Best fit model d.f. = 7,13		Whole model d.f. = 11,9		Best fit model d.f. = 8,14		Whole model d.f. = 11,9		Best fit model d.f. = 8,14	
	F-value	P	F-value	P	F-value	P	F-value	P	F-value	P	F-value	P
**Fire Severity**	16.41	(-)***	22.33	(-)***	10.81	(+)**	15.20	(+)***	0.32	(+)	0.17	(+)
**Foliage Cover**	0.26	(-)	0.35	(-)	0.01	(+)	0.01	(+)	0.16	(+)		
**Bare Soil**	2.59	(-)	3.52	(-).	5.56	(+)*	7.81	(+)*	2.87	(+)	4.25	(+)*
**Moss Cover**	0.54	(-)	0.74	(-)	0.02	(-)			0.00	(+)		
**Litter Cover**	0.20	(+)			2.06	(-)			0.06	(+)		
**Litter Depth**	1.08	(-)			0.01	(+)			2.12	(-)		
**Soil disturbance**	0.44	(+)	0.47	(+)	0.99	(+)			0.55	(-)		
**Moisture**	0.24	(+)			8.28	(-)*	10.02	(-)**	6.56	(-)*	12.39	(-)**
**Relative Humidity**	0.30	(-)			0.81	(-)	1.76	(-)	1.36	(-)	2.33	(-)
**Distance to Log**	3.20	(+)	6.2	(+)*	0.09	(+)			0.61	(+)		

In terms of microhabitat variables, the size and biomass of macroinvertebrate detritivores had a highly significant negative relationship with moisture and was significantly positively related to percentage of bare soil. Neither moisture, nor bare soil affected detritivore abundance. Macroinvertebrate detritivores were more abundant at increasing distances from logs. Despite, strong effects of fire severity on macroinvertebrate traits, there were no other microhabitat measures that served as significant predictors of abundance, size or biomass (Table 3).

## Discussion

We addressed the poorly studied question of how fire severity affects macroinvertebrate detritivore assemblage characteristics and function, to gain insights into the potential impact of fire severity on litter build-up. Litter accumulation enhances the likelihood of fires, so it is important to understand its relationship with fire severity if we are to manage fires. Our study showed that macroinvertebrate detritivore assemblages were changed by fire severity, with larger but less abundant macroinvertebrates under high fire severity. Exclusion of macroinvertebrate detritivores from litter demonstrated their substantial functional importance within this system. However, we detected little differentiation in the function of macroinvertebrate detritivores among fire severity classes, despite significant changes in microhabitat conditions after severe wildfire, suggesting that the functional effects of fire severity may have been short-lived. We suggest that this resulted from functional redundancy in the macroinvertebrate detritivore assemblage, whereby a low abundance of large detritivores in crown burnt sites resulted in equivalent decomposition rates to those in less severely burnt sites, where detritivores were smaller, but more abundant.

### The effects of fire severity on macroinvertebrate detritivore size

Although macroinvertebrate assemblages differed among fire severity classes, with higher abundance and smaller individuals in low severity classes, macroinvertebrate biomass and function were similar. We propose two possible explanations for differences in body size in response to fire severity: 1) body size affects the ability to recolonise; and 2) post-fire microhabitats favour large body size.

Firstly, larger insect species are better active dispersers (e.g., Lepidoptera, [62,63], Coleoptera, [64]). Large body size improves dispersal ability because it reduces the mass-specific cost of flight [65,66]. It is also thought to improve dispersal ability by enhancing resistance to starvation [67]. Among flightless species, large body size might also enhance dispersal if allometric scaling results in relatively longer legs and because desiccation tolerance (and therefore the variety of microhabitats through which a species can pass) increases with body size. However, previous studies have failed to find a link between the body size of beetles and their response to large-scale disturbances such as habitat fragmentation [68–70].

Alternatively, differences in size may result from macroinvertebrate detritivore assemblage responses to microhabitat changes following fires, including soil disturbance and moss cover. Changes in microhabitats bring challenges related to desiccation, food resource availability and physical mobility. Moisture was a strong predictor of size: sites with lower moisture levels supported larger individuals. High severity fires remove foliage cover, leaf litter and humus in surface soil, ultimately leading to greater temperature and moisture fluctuation [27,71]. In simplified habitats, larger body size will reduce moisture loss as a result of lower surface area to volume ratios [72]. Additionally, larger species have been shown to better utilize bulky food items of low resource quality [73,74] including *Eucalyptus* leaves [75]. Macroinvertebrate detritivores of smaller body size are better able to access the resulting finer scale resources [27], common in unburnt sites. The loss of fragmented and partially decomposed leaf litter at crown burnt sites should favour macroinvertebrate detritivores of large body size able to maintain function by exploiting whole recently fallen leaves. Previous studies also suggest that structurally simplified habitats support larger species (e.g. [76–78]) because complex habitats may act as an impediment to movement. Greater dominance of larger species following fire may even result in an acceleration of functions due to their greater mobility [79].

### The effects of fire severity on the function of macroinvertebrate detritivores

Macroinvertebrate exclusion reduced litter mass loss rates by 34.7%. This is in agreement with a global average of 35% [26], but is higher than the 21% average recorded for deciduous forest, which is closest in latitude to the study area. Mass loss for the insecticide treatment is attributed to chemical leaching and activity of microbes [28,80]. However, microinvertebrates were present at all sites for the insecticide treatment, (approximately 80% reduction). Microinvertebrates include fungivores (collembolans and mites) that graze on fungal hyphae that may alter the function and identity of microbes [40]. There was no mass loss difference between fire severities (insecticide treatments). However, due the substantial reduction of microinvertebrate fungivores, we cannot rule out the possibility of potential indirect effects on decomposition.

A key finding of this study was that macroinvertebrate detritivore function was not inhibited by high fire severity, either in terms of loss of leaf litter or variation in litter loss. This is despite distinct differences in the body size and abundance of macroinvertebrates between fire severity classes. A trade-off in body size and abundance resulted in similar macroinvertebrate biomass across fire severity classes, which was directly reflected in similarities in the rate of litter breakdown. Disturbance can affect ecosystem function [81]. However, in this study, disturbance caused by high severity fire resulted in an assemblage with species that differed in an important trait (body size), but had similar functional effects. Leaf litter breakdown is a major component of annual energy flow and nutrient cycling within forest ecosystems [82] and functional redundancy in macroinvertebrate detritivores (and therefore species diversity) is likely to have played an important role in maintaining this process under changed conditions. Our study shows that macroinvertebrate detritivore function can be maintained independent of fire severity and may thus prevent positive feedback driving increased fire disturbance.

### Management implications

Leaf litter contributes to the spread of wildfire and forest managers aim to monitor and reduce this component of the fuel load through planned fuel reduction burns [83]. We showed that litter depth and decomposition rate three years after fire is similar in sites experiencing different fire severities. We thus suggest that, within three years after fire, low severity burns, such as those performed for fuel reduction, neither promote nor decrease litter decomposition and therefore the likelihood of future fires in this forest type. Litter decomposition remains similar even following severe fires, suggesting that there is no feedback promoting future severe fires through changes to litter decomposition. No studies have investigated the effects of fire severity on litter decomposition in other forest types, so further work is required to determine the generality of these findings. In this system, macroinvertebrate detritivore assemblages responsible for decomposition appear to have a high level of functional resilience to fire severity, which we attribute to rapid recovery of a decomposer assemblage of large body size. This resilience is likely to be a result of the fire prone nature of the system, with species adapted to a periodic occurrence of severe fires. Prescribed fuel reduction burns based on fuel loads has meant a three year fire interval has been considered possible in southern Australian forests where reducing fire risk around property is a high priority [45,84]. Although we have shown functional resilience to fire severity, repeated fires may slow decomposition rates substantially [36] and their effectiveness in diminishing fuel loads will be marginal unless fires are very frequent [83].

## Supporting Information

S1 Fig Map of the 2009 Kilmore-Murrindindi fire complex study area with 21 sites (three fire severities: Crown Burnt, Ground Burnt and Unburnt). (TIF)

S1 Table Site co-ordinates (UTM, WGS84), aspect and slope. (TIF)

S2 Table Raw Data 5 Mar 2015. (TIF)
