# Supplementary figures and images for: The Effects of Fire Severity on Macroinvertebrate Detritivores and Leaf Litter Decomposition
Source: PLoS One. 2015 Apr 16;10(4):e0124556. doi: 10.1371/journal.pone.0124556 (PMC4399839; doi:10.1371/journal.pone.0124556)

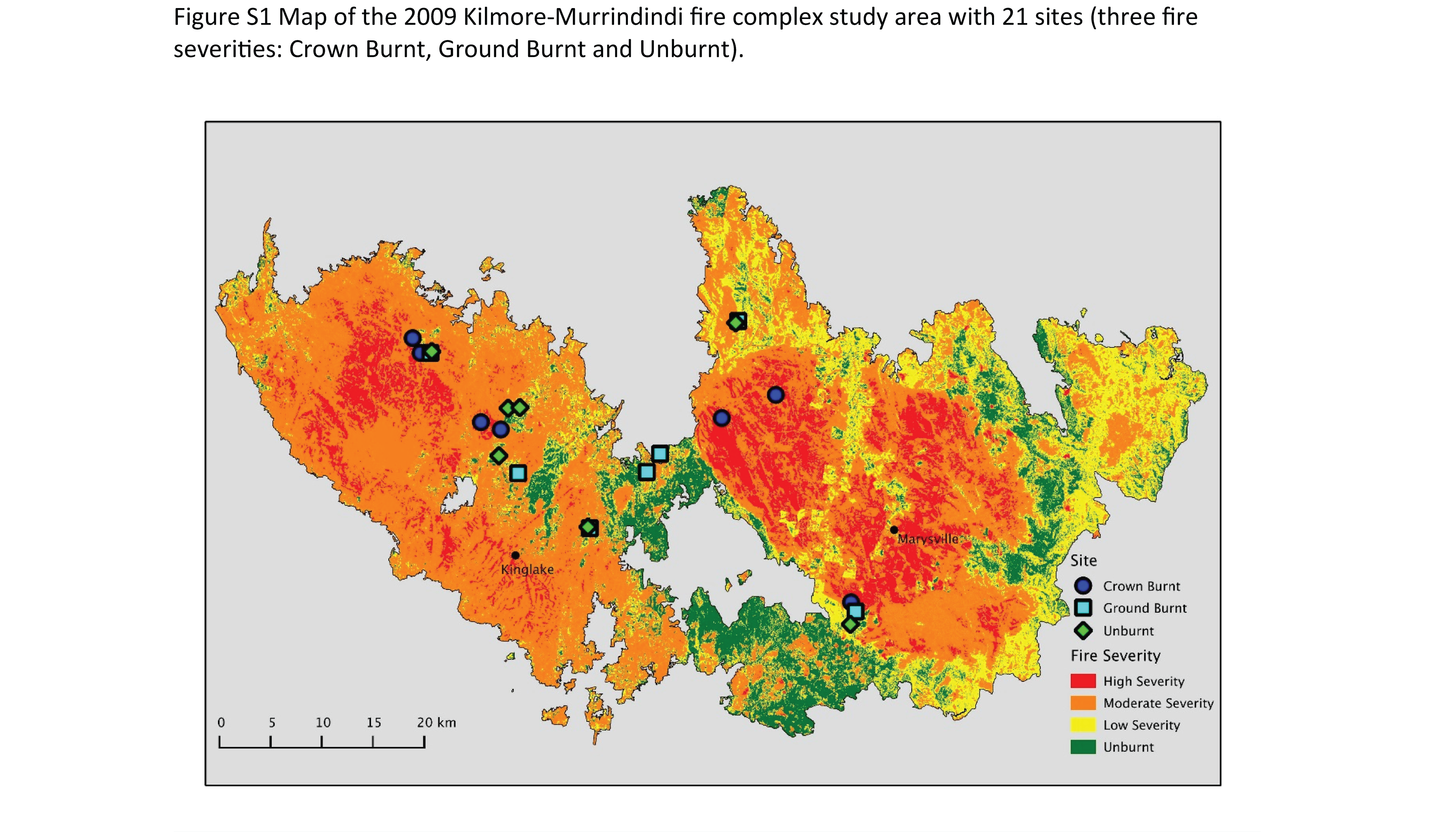

Supplement: S1 Fig — (TIF) [file pone.0124556.s001.tif]

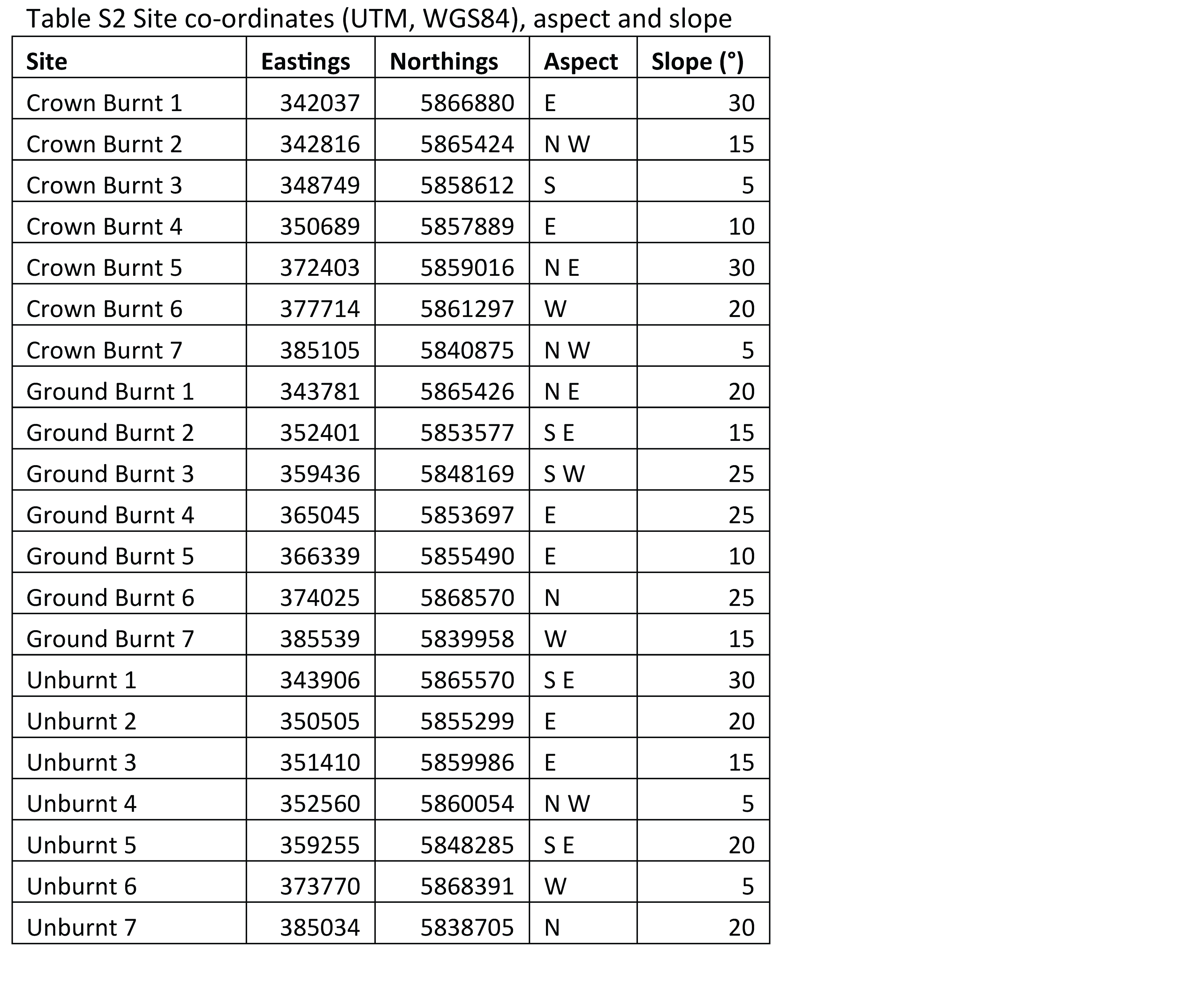

Supplement: S1 Table — (TIF) [file pone.0124556.s002.tif]

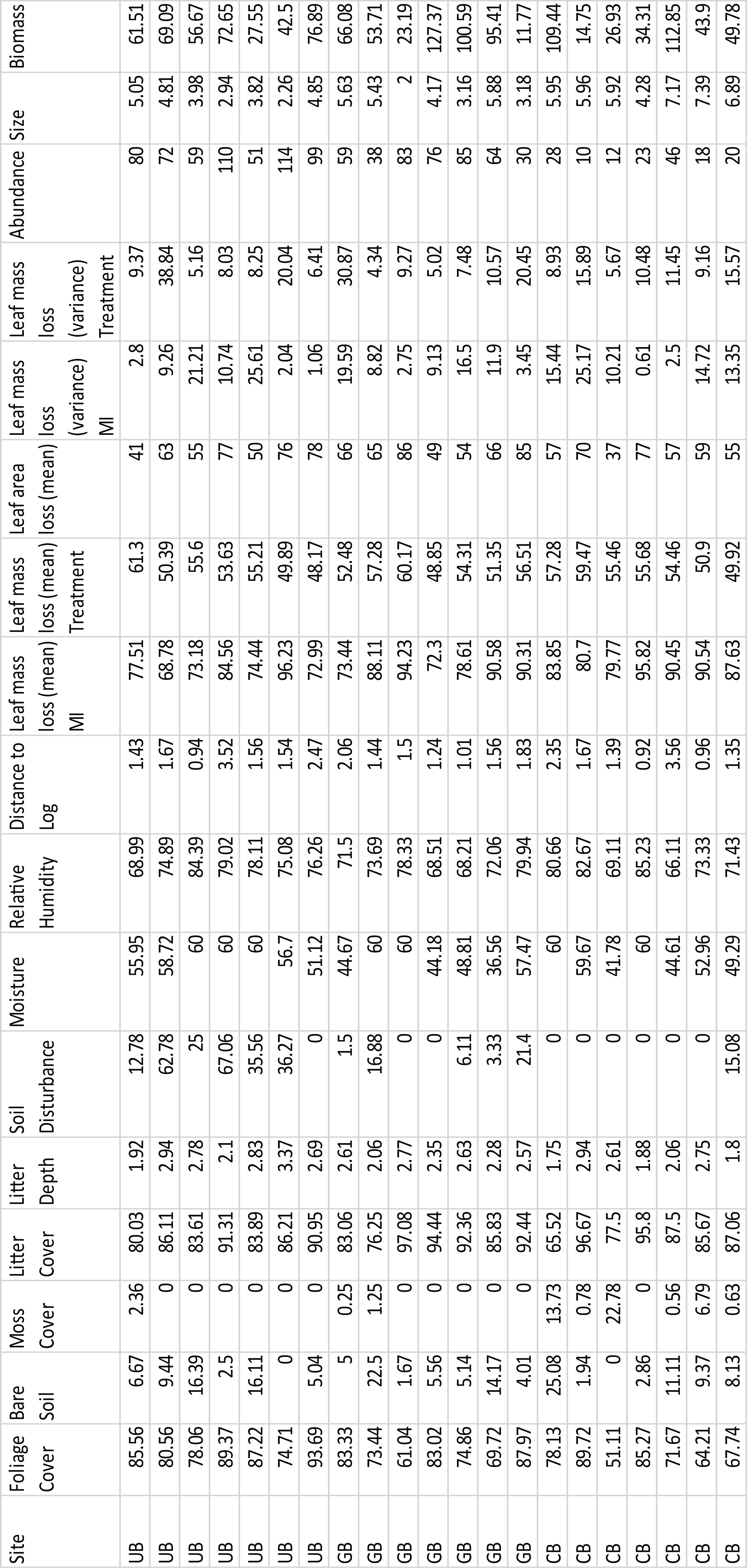

Supplement: S2 Table — (TIF) [file pone.0124556.s003.tif]
